# Supplementary material for: CaMKII oxidation is a critical performance/disease trade-off acquired at the dawn of vertebrate evolution
Source: Nat Commun. 2021 May 26;12:3175. doi: 10.1038/s41467-021-23549-3 (PMC8155201; doi:10.1038/s41467-021-23549-3)
Supplement: Supplementary file 1 — Supplementary Information [file 41467_2021_23549_MOESM1_ESM.pdf]

Supplementary Information for

CaMKII oxidation is a critical performance/disease trade-off acquired at the dawn of vertebrate evolution

Wang et al.

This PDF file includes:

Supplementary Figures 1-16

Supplementary Tables 1-4

Supplementary References

Non-Vertebrate Eukaryota	Monosiga_brevicollis_(Choanoflagellate)_(A9UZT1)	A	L	K	H	P	W	L	A	D	A	S	-	V	P	S	T	V	H	R	Q	T	T	I	D	E	L	Q	R	F	N	A	R	R	K	L	K	G	S	V	K	A	V	M	G	V	G	R	M	L
	Salpingoeca_rosetta_(Choanoflagellate)_(F2UPG5)	L	L	Q	H	P	W	I	A	R	R	D	-	V	P	G	S	V	H	R	Q	A	T	L	E	E	L	K	K	F	N	A	R	R	K	L	K	G	G	V	N	A	V	I	G	V	S	K	M	M
	Capsaspora_owczarzaki_(Filasterea)_(A0A0D2US79)	A	L	A	H	P	W	I	A	Q	S	D	R	V	A	P	H	V	H	R	Q	G	T	I	E	E	M	K	R	F	N	A	R	R	K	F	K	G	A	I	F	T	A	I	A	T	N	K	L	L
	Trichoplax_adhaerens_(Placozoa)_(B3S632)	A	L	K	H	P	F	I	L	N	R	D	N	V	C	A	K	V	H	R	Q	K	T	I	D	G	L	R	R	F	N	A	R	R	K	L	K	G	A	I	L	T	T	I	L	A	T	R	T	L
	Suberites_domuncula_(Sponge)_(Q9U5L0)	A	L	D	H	T	W	I	K	N	C	D	R	V	A	S	M	V	H	R	Q	E	T	I	V	V	L	R	K	F	N	A	R	R	K	L	K	A	A	V	H	T	A	L	L	V	T	K	R	S
	Hydra_vulgari_(T2M388)	A	L	K	H	P	W	I	S	D	R	H	A	C	A	S	T	F	H	R	Q	E	T	I	N	G	L	K	R	F	N	A	R	R	K	L	K	G	A	I	L	T	T	V	F	A	R	R	I	S
	Nematostella_vectensis_(starlet_sea_anemone)_(A7RF52)	A	L	K	H	P	W	I	V	N	R	E	R	I	A	S	K	V	H	R	Q	Q	T	L	D	G	L	K	R	F	N	A	R	R	K	L	R	G	A	I	H	A	T	I	L	A	T	H	N	F
	Nematostella_vectensis_(starlet_sea_anemone)_(A7T0H5)	A	L	K	H	P	W	I	V	N	R	E	R	V	A	S	K	V	H	R	Q	E	T	L	D	G	L	K	K	F	N	A	R	R	K	L	K	G	A	I	L	T	T	I	L	A	T	S	N	F
	CaMKII_(Orchesella_cincta_springtail_A0A1D2NGA4)	A	L	K	H	P	W	I	C	Q	R	E	R	V	A	S	L	V	H	R	Q	E	T	V	D	C	L	K	K	F	N	A	R	R	K	L	K	G	A	I	L	T	T	M	L	A	T	R	N	F
	CaMKII_(Periplaneta_americana_American_cockroach_H6SWV0)	A	L	K	H	P	W	I	C	Q	R	E	R	V	A	S	V	V	H	R	Q	E	T	V	D	C	L	K	K	F	N	A	R	R	K	L	K	G	A	I	L	T	T	M	L	A	T	R	N	F
	CaMKII_(Scylla_olivacea_A0A0P4WI42)	A	L	K	H	P	W	I	C	Q	R	E	R	V	A	S	V	V	H	R	Q	E	T	V	D	C	L	R	K	F	N	A	R	R	K	L	K	S	A	I	L	T	T	M	L	A	T	R	N	F
	CaMKII_(Lygus_hesperus_Western_plant_bug_A0A0A9WPF4)	A	L	K	H	P	W	I	C	Q	R	E	R	V	A	S	V	V	H	R	Q	E	T	V	D	C	L	K	K	F	N	A	R	R	K	L	K	G	A	I	L	T	T	M	L	A	T	R	N	F
	CaMKII_(Daphnia_pulex_water_flea_E9GTA8)	A	L	K	H	P	W	I	C	Q	R	E	R	V	A	S	V	V	H	R	Q	E	T	V	D	C	L	K	K	F	N	A	R	R	K	L	K	G	A	I	L	T	T	M	L	A	T	R	N	F
	CaMKII_(Strigamia_maritima__T1INT6)	A	L	K	H	P	W	I	C	Q	R	E	R	V	A	S	V	V	H	R	Q	E	T	V	D	C	L	K	K	F	N	A	R	R	K	L	K	G	A	I	L	T	T	M	L	A	T	R	N	F
	CaMKII_(Drosophila_melanogaster_Q00168)	A	L	K	H	P	W	I	C	Q	R	E	R	V	A	S	V	V	H	R	Q	E	T	V	D	C	L	K	K	F	N	A	R	R	K	L	K	G	A	I	L	T	T	M	L	A	T	R	N	F
	CaMKII_(Cupiennius_salei_American_Wandering_spider_T1DG40)	A	L	K	H	P	W	I	C	Q	R	E	R	V	A	S	T	L	H	R	Q	E	T	V	D	C	L	K	K	F	N	A	R	R	K	L	K	G	A	I	L	T	T	M	L	A	T	R	N	F
	CaMKII_type_2_(Cupiennius_salei_American_Wandering_spider_T	A	L	K	H	P	W	I	C	Q	R	E	R	V	A	S	T	L	H	R	Q	E	T	V	E	C	L	K	K	F	N	A	R	R	K	L	K	G	A	I	L	T	T	M	L	A	T	R	N	F
	CaMKII_(Limulus_polyphemus_(Atlantic_horseshoe_crab)_Q94608	A	L	K	H	P	W	I	C	Q	R	E	R	V	A	S	T	L	H	R	Q	E	T	V	E	C	L	K	K	F	N	A	R	R	K	L	K	G	A	I	L	T	T	M	L	A	T	R	N	F
	CaMKII_(Caenorhabditis_elegans_O62305)	A	L	K	V	P	W	I	C	N	R	E	R	V	A	S	A	I	H	R	Q	D	T	V	D	C	L	K	K	F	N	A	R	R	K	L	K	A	A	I	S	A	V	K	M	V	T	R	M	S
	Strongylocentrotus_purpuratus_(Q6UVK0)	A	L	K	H	P	W	I	Q	N	R	D	R	Y	A	S	A	M	H	R	Q	E	T	V	D	C	L	K	K	F	N	A	R	R	K	L	K	G	A	I	L	T	T	M	L	A	T	R	N	F
	CaMKII_(Crassostrea_gigas_K1PZN1)	A	L	K	H	P	W	I	C	Q	R	E	R	V	A	S	V	V	H	R	Q	E	T	V	D	C	L	K	K	F	N	A	R	R	K	L	K	G	A	I	L	T	T	M	L	A	T	R	N	F
	CaMKII_(Capitella_teleta_R7V081)	A	L	K	H	P	W	I	C	Q	R	E	R	V	A	S	M	V	H	R	Q	E	T	V	D	C	L	K	K	F	N	A	R	R	K	L	K	G	A	I	L	T	T	M	L	V	S	R	N	F
	Ciona_intestinalis_(A0A1W2W5K0)	A	L	R	N	P	W	I	C	Q	R	D	R	V	A	S	H	I	H	R	Q	D	T	V	D	C	L	K	T	F	N	A	R	R	K	L	K	G	A	I	I	T	T	M	L	A	T	R	N	F
	Branchiostoma_floridae_(Lancelet)_(cDNA_clone_CAXC6708_5)	A	L	R	H	P	W	I	C	Q	R	E	R	V	A	S	Q	I	H	R	Q	E	T	V	D	C	L	K	K	F	N	A	R	R	K	L	K	G	A	I	L	T	T	M	L	A	T	R	N	F
Vertebrates	Petromyzon_marinus_putative_CaMKIIa_(Lamprey)_(S4RND2)	A	L	T	H	P	W	I	F	Q	R	S	T	V	A	S	C	M	H	R	Q	E	T	V	E	C	L	K	K	F	N	A	R	R	K	L	K	G	A	I	L	T	T	M	L	A	T	R	N	F
	Hagfish_CaMKII_(partial)_translated_from_reverse_complementa	A	L	K	H	P	W	I	C	Q	R	S	T	V	A	S	C	M	H	R	Q	E	T	V	D	C	L	K	K	F	N	A	R	R	K	L	K	G	A	I	L	T	T	M	L	A	T	R	N	F
	Callorhinchus_milii_CaMKIIa_(V9KL89)	V	L	K	H	P	W	I	S	H	R	A	T	V	A	S	C	M	H	R	Q	E	T	V	D	C	L	K	K	F	N	A	R	R	K	L	K	G	A	I	L	T	T	M	L	A	T	R	N	F
	Callorhinchus_milii__CaMKIIb_(V9KB81)	A	L	K	H	P	W	I	C	Q	R	S	T	V	A	S	C	M	H	R	Q	E	T	V	E	C	L	K	K	F	N	A	R	R	K	L	K	G	A	I	L	T	T	M	L	A	T	R	N	F
	Callorhinchus_milii_CaMKIId_(V9KAW6)	A	L	K	H	P	W	I	C	Q	R	S	T	V	A	S	C	M	H	R	Q	E	T	V	E	C	L	K	K	F	N	A	R	R	K	L	K	G	A	I	L	T	T	M	L	A	T	R	N	F
	Callorhinchus_milii_CaMKIIg_(V9K7V2)	A	L	K	H	P	W	V	C	Q	R	S	T	V	A	S	M	M	H	R	Q	E	T	V	E	C	L	R	K	F	N	A	R	R	K	L	K	G	A	I	L	T	T	M	L	A	T	R	N	F
	CaMKIIg1_Danio_rerio_(Zebrafish)_(A0A0R4IUL7)	A	L	K	H	P	W	V	C	Q	R	S	T	V	A	S	M	M	H	R	Q	E	T	V	E	C	L	R	K	F	N	A	R	R	K	L	K	G	A	I	L	T	T	M	L	V	S	R	N	F
	CaMKIIg2_Danio_rerio_(Zebrafish)_(A0A0R4IQT3)	A	L	K	H	P	W	V	C	Q	R	S	T	V	A	S	M	M	H	R	Q	E	T	V	E	C	L	R	K	F	N	A	R	R	K	L	K	G	A	I	L	T	T	M	L	V	S	R	N	F
	CaMKIIb1_Danio_rerio_(Zebrafish)_(A2BGW3)	A	L	K	H	P	W	V	C	Q	R	S	T	V	A	S	M	M	H	R	Q	E	T	V	E	C	L	K	K	F	N	A	R	R	K	L	K	G	A	I	L	T	T	M	L	V	S	R	N	F
	CaMKIIb2_Danio_rerio_(Zebrafish)_(A0A286Y8I7)	A	L	K	H	P	W	V	C	Q	R	S	T	V	A	S	M	M	H	R	Q	E	T	V	E	C	L	K	K	F	N	A	R	R	K	L	K	G	A	I	L	T	T	M	L	V	S	R	N	F
	CaMKIIa_Danio_rerio_(Zebrafish)_(A0A286Y9M9)	A	L	K	H	P	W	I	S	H	R	S	T	V	A	S	C	M	H	R	Q	E	T	V	E	C	L	K	K	F	N	A	R	R	K	L	K	G	A	I	L	T	T	M	L	A	T	R	N	F
	CaMKIId1_Danio_rerio_(Zebrafish)_(Q6DEH3)	A	L	K	H	P	W	I	C	Q	R	S	T	V	A	S	M	M	H	R	Q	E	T	V	E	C	L	K	K	F	N	A	R	R	K	L	K	G	A	I	L	T	T	M	L	A	T	R	N	F
	CaMKIId2_Danio_rerio_(Zebrafish)_(Q6DGS3)	A	P	K	H	P	W	I	C	Q	R	S	T	V	A	S	M	M	H	R	Q	E	T	V	E	C	L	K	K	F	N	A	R	R	K	L	K	G	A	I	L	T	T	L	L	V	T	R	N	F
	CaMKIIa_(Lepisosteus_oculatus_(Spotted_gar)_W5MZ72)	A	L	K	H	P	W	I	S	H	R	S	T	V	A	S	C	M	H	R	Q	E	T	V	D	C	L	K	K	F	N	A	R	R	K	L	K	G	A	I	L	T	T	M	L	A	T	R	N	F
	CaMKIIg_(Lepisosteus_oculatus_(Spotted_gar)_W5N4B9)	A	L	K	H	P	W	V	C	Q	R	S	T	V	A	S	M	M	H	R	Q	E	T	V	E	C	L	R	K	F	N	A	R	R	K	L	K	G	A	I	L	T	T	M	L	V	S	R	N	F
	CaMKIId_(Lepisosteus_oculatus_(Spotted_gar)_W5N6B1)	A	L	K	H	P	W	I	C	Q	R	S	T	V	A	S	M	M	H	R	Q	E	T	V	E	C	L	K	K	F	N	A	R	R	K	L	K	G	A	I	L	T	T	M	L	A	T	R	N	F
	CaMKIIb_(Lepisosteus_oculatus_(Spotted_gar)_W5NDW9)	A	L	K	H	P	W	V	C	Q	R	S	T	V	A	S	M	M	H	R	Q	E	T	V	E	C	L	K	K	F	N	A	R	R	K	L	K	G	A	I	L	T	T	M	L	A	T	R	N	F
	CaMKIIg_(Latimeria_chalumnae_H3AV19)	A	L	K	H	P	W	V	C	Q	R	S	T	V	A	S	M	M	H	R	Q	E	T	V	E	C	L	R	K	F	N	A	R	R	K	L	K	G	A	I	L	T	T	M	L	V	S	R	N	F
	CaMKIIb_(Latimeria_chalumnae_H3AVR3)	A	L	R	H	P	W	V	C	Q	R	S	T	V	A	S	M	M	H	R	Q	E	T	V	E	C	L	K	K	F	N	A	R	R	K	L	K	G	A	I	L	T	T	M	L	A	T	R	N	F
	CaMKIIa_(Latimeria_chalumnae_H3B5X6)	V	L	K	H	P	W	I	S	H	R	S	T	V	A	S	C	M	H	R	Q	E	T	V	D	C	L	K	K	F	N	A	R	R	K	L	K	G	A	I	L	T	T	M	L	A	T	R	N	F
	CaMKIIa_(Xenopus_laevis_A0A1L8GRD5)	A	L	K	H	P	W	I	S	H	R	S	T	V	A	S	C	M	H	R	Q	E	T	V	D	C	L	K	K	F	N	A	R	R	K	L	K	G	A	I	L	T	T	M	L	A	T	R	N	F
	CaMKIIb_(Xenopus_laevis_Q6GLR3)	A	L	K	H	P	W	V	C	Q	R	S	T	V	A	S	M	M	H	R	Q	E	T	V	E	C	L	K	K	F	N	A	R	R	K	L	K	G	A	I	L	T	T	M	L	A	T	R	N	F
	CaMKIIg_(Xenopus_laevis_A0A1L8FJZ5)	A	L	K	H	P	W	V	C	Q	R	S	T	V	A	S	M	M	H	R	Q	E	T	V	E	C	L	R	K	F	N	A	R	R	K	L	K	G	A	I	L	T	T	M	L	V	S	R	N	F
	CaMKIId_(Xenopus_laevis_A0A1L8HUU6)	A	L	R	H	P	W	I	C	Q	R	S	T	V	A	S	M	M	H	R	Q	E	T	V	D	C	L	K	K	F	N	A	R	R	K	L	K	G	A	I	L	T	T	M	L	A	T	R	N	F
	CaMKIIa_(Gallus_gallus_F1NGS8)	A	L	K	H	P	W	I	S	H	R	A	T	V	A	S	C	M	H	R	Q	E	T	V	D	C	L	K	K	F	N	A	R	R	K	L	K	G	A	I	L	T	T	M	L	A	T	R	N	F
	CaMKIIb_(Gallus_gallus_O93560)	A	L	K	H	P	W	V	C	Q	R	S	T	V	A	S	M	M	H	R	Q	E	T	V	E	C	L	K	K	F	N	A	R	R	K	L	K	G	A	I	L	T	T	M	L	A	T	R	N	F
	CaMKIIg_(Gallus_gallus_F1P1J7)	A	L	K	H	P	W	V	C	Q	R	S	T	V	A	S	M	M	H	R	Q	E	T	V	E	C	L	R	K	F	N	A	R	R	K	L	K	G	A	I	L	T	T	M	L	V	S	R	N	F
	CaMKIId_(Gallus_gallus_A0A1D5NZG7)	A	L	K	H	P	W	I	C	Q	R	S	T	V	A	S	M	M	H	R	Q	E	T	V	D	C	L	K	K	F	N	A	R	R	K	L	K	G	A	I	L	T	T	M	L	A	T	R	N	F
	CaMKIIa_(Alligator_mississippiensis_A0A151M7C2)	A	L	K	H	P	W	I	S	H	R	A	T	V	A	S	C	M	H	R	Q	E	T	V	D	C	L	K	K	F	N	A	R	R	K	L	K	G	A	I	L	T	T	M	L	A	T	R	N	F
	CaMKIIb_(Alligator_mississippiensisA0A151MMF3)	A	L	K	H	P	W	V	C	Q	R	S	T	V	A	S	M	M	H	R	Q	E	T	V	E	C	L	K	K	F	N	A	R	R	K	L	K	G	A	I	L	T	T	M	L	A	T	R	N	F
	CaMKIIg_(Alligator_mississippiensis_A0A151MSW0)	A	L	K	H	P	W	V	C	Q	R	S	T	V	A	S	M	M	H	R	Q	E	T	V	E	C	L	R	K	F	N	A	R	R	K	L	K	G	A	I	L	T	T	M	L	V	S	R	N	F
	CaMKIId_(Alligator_mississippiensis_A0A151PEV2)	A	L	K	H	P	W	I	C	Q	R	S	T	V	A	S	M	M	H	R	Q	E	T	V	D	C	L	K	K	F	N	A	R	R	K	L	K	G	A	I	L	T	T	M	L	A	T	R	N	F
	CaMKIIa_(Anolis_carolinesis_L7MZY1)	A	L	K	H	P	W	I	S	H	R	S	T	V	A	S	C	M	H	R	Q	E	T	V	D	C	L	K	K	F	N	A	R	R	K	L	K	G	A	I	L	T	T	M	L	A	T	R	N	F
	CaMKIIb_(Anolis_carolinesis_H9GIS3)	A	L	K	H	P	W	V	C	Q	R	S	T	V	A	S	M	M	H	R	Q	E	T	V	E	C	L	K	K	F	N	A	R	R	K	L	K	G	A	I	L	T	T	M	L	A	T	R	N	F
	CaMKIId_(Anolis_carolinesis_L7MZX9)	A	L	K	H	P	W	I	C	Q	R	S	T	V	A	S	M	M	H	R	Q	E	T	V	D	C	L	K	K	F	N	A	R	R	K	L	K	G	A	I	L	T	T	M	L	A	T	R	N	F
	caMKIIg_(Anolis_carolinensis_H9GF19)	A	L	K	H	P	W	V	C	Q	R	S	T	V	A	S	M	M	H	R	Q	E	T	V	E	C	L	R	K	F	N	A	R	R	K	L	K	G	A	I	L	T	T	M	L	V	S	R	N	F
	CaMKIIa_(Mus_musculus_P11798)	A	L	K	H	P	W	I	S	H	R	S	T	V	A	S	C	M	H	R	Q	E	T	V	D	C	L	K	K	F	N	A	R	R	K	L	K	G	A	I	L	T	T	M	L	A	T	R	N	F
	CaMKIIb_(Mus_musculus_P28652)	A	L	K	H	P	W	V	C	Q	R	S	T	V	A	S	M	M	H	R	Q	E	T	V	E	C	L	K	K	F	N	A	R	R	K	L	K	G	A	I	L	T	T	M	L	A	T	R	N	F
	CaMKIId_(Mus_musculus_Q6PHZ2)	A	L	K	H	P	W	I	C	Q	R	S	T	V	A	S	M	M	H	R	Q	E	T	V	D	C	L	K	K	F	N	A	R	R	K	L	K	G	A	I	L	T	T	M	L	A	T	R	N	F
	CaMKIIg_(Mus_musculus_Q923T9)	A	L	K	H	P	W	V	C	Q	R	S	T	V	A	S	M	M	H	R	Q	E	T	V	E	C	L	R	K	F	N	A	R	R	K	L	K	G	A	I	L	T	T	M	L	V	S	R	N	F
	CaMKIIb_(Homo_sapiens_Q13554)	A	L	K	H	P	W	V	C	Q	R	S	T	V	A	S	M	M	H	R	Q	E	T	V	E	C	L	K	K	F	N	A	R	R	K	L	K	G	A	I	L	T	T	M	L	A	T	R	N	F
	CaMKIIg_(Homo_sapiens_Q13555)	A	L	K	H	P	W	V	C	Q	R	S	T	V	A	S	M	M	H	R	Q	E	T	V	E	C	L	R	K	F	N	A	R	R	K	L	K	G	A	I	L	T	T	M	L	V	S	R	N	F
	CaMKIId_(Homo_sapiens_Q13557)	A	L	K	H	P	W	I	C	Q	R	S	T	V	A	S	M	M	H	R	Q	E	T	V	D	C	L	K	K	F	N	A	R	R	K	L	K	G	A	I	L	T	T	M	L	A	T	R	N	F
	CaMKIIa_(Homo_sapiens_Q9UQM7)	A	L	K	H	P	W	I	S	H	R	S	T	V	A	S	C	M	H	R	Q	E	T	V	D	C	L	K	K	F	N	A	R	R	K	L	K	G	A	I	L	T	T	M	L	A	T	R	N	F

Supplementary Fig. 1 Sequence alignment of CaMKII regulatory domains reveals the emergence and conservation of ox-CaMKII in vertebrates. The paired oxidation sensitive amino acid residues (CM and MM at positions corresponding to residues 281 and 282 of mouse CaMKIIγ, highlighted yellow) are required for ox-CaMKII and are present only in the vertebrates in the above examples. In contrast, none of the sampled invertebrate CaMKII above possess the MM/CM pair (highlighted in light blue). For a comprehensive summary and exceptions see Supplementary Table 1.

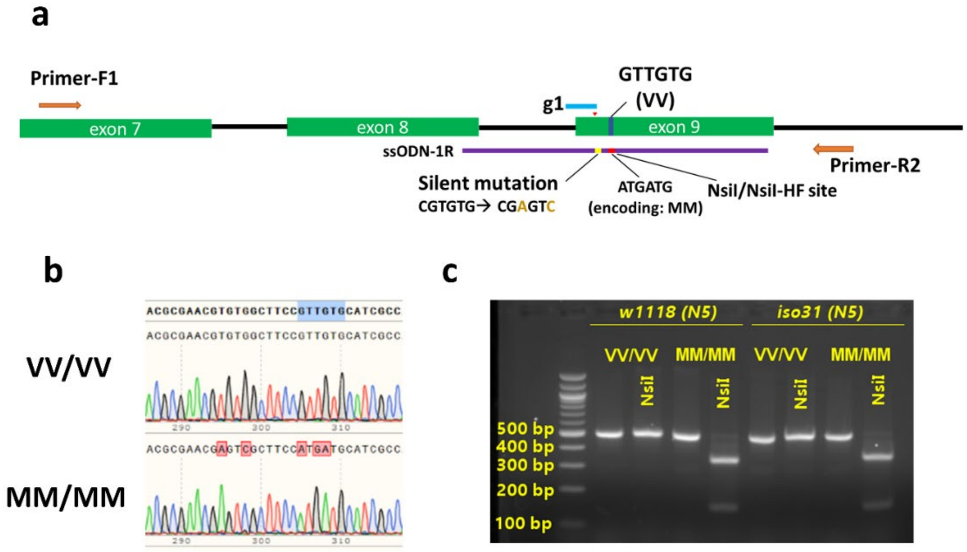

Supplementary Fig. 2 Generation of *CaMKII*^{MM}/*CaMKII*^{MM} flies using CRISPR. a Schematics of the CRISPR guide designs and the single strand template (ssODN-1R) that mediated the homology directed recombination, resulting in mutations of codons from encoding VV to encoding MM, and introduction of the silent mutations for NsiI/NsiI-HF restriction site. Primers-F1/R2 anneal outside of the range homologous to the ssODN-1R to amplify the genomic region for genotyping. **b** Chromatograms of sequencing results from the PCR products amplified by primers F1 and R2 from homozygous VV/VV (top) and MM/MM (bottom) flies. The VV/VV (wild type) and MM/MM flies are referred to as VV and MM flies in the text for brevity. **c** Agarose gel electrophoresis of PCR products from flies backcrossed into the *w1118* or *iso31* genetic background for 5 generations. The genotypes of flies were determined by digesting the 493 bp PCR products with the restriction enzyme NsiI. PCR products amplified from the VV allele were resistant to NsiI, while those from the MM allele were cut into 345 bp and 148 bp fragments. The gel picture is representative of 6 independent experiments. Source data are provided in a Source Data file.

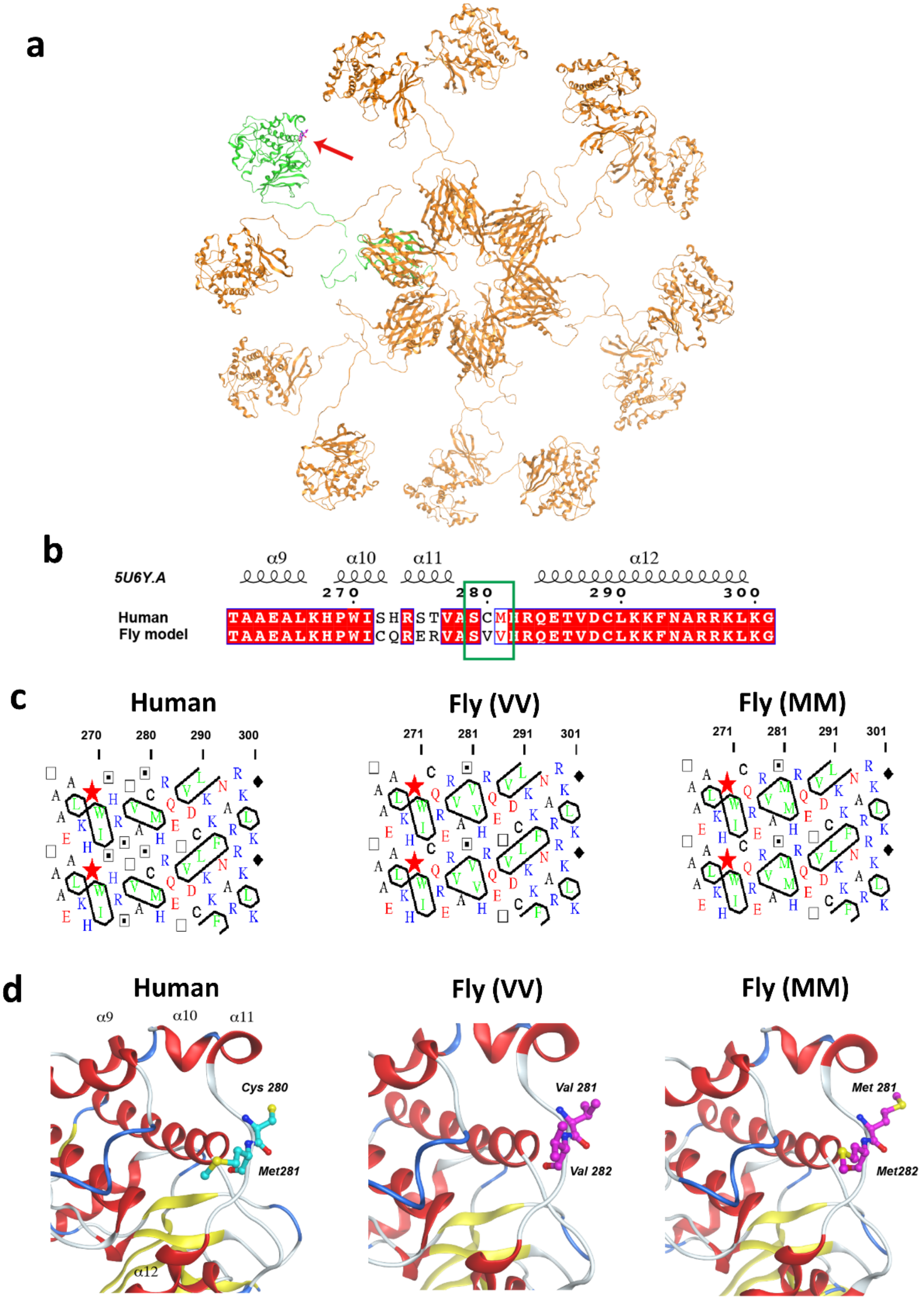

Supplementary Fig. 3 Modeling the impact of MM to VV mutation in fly CaMKII. a Comparative model of *Drosophila melanogaster* CaMKII based on the human CaMKIIα. The dodecameric enzyme is shown with a monomer-colored green. The red arrow points to the fly VV residues. The model predicts that such residues are exposed and appears not to participate in any intermonomer contacts. **b** Sequence alignment between template and target of the

comparative model. The sequence around the region of interest (green box) is shown with equivalent residues in red boxes and residues of similar characteristics with red characters in white boxes. The corresponding secondary structure observed in the crystal structure (5U6Y [10.2210/pdb5U6Y/pdb]) is drawn on top of the alignment as coils representing α-helices. **c** Hydrophobic cluster analysis of the region. The hydrophobic cluster analysis performed using the HCA server (http://bioserv.rpbs.univ-paris-diderot.fr/services/HCA/) shows conservation of the characteristics among human, fly VV- and fly MM-CaMKII. The protein sequences are represented as a bidimensional alpha-helicoidal plot, which has been shown to offer the best correspondence between hydrophobic clusters and regular secondary structures[1-3]. Hydrophobic residues M, L, I, V, F, Y, W are in green and cluster together if they are separated from the cluster members for less than four residues in the sequence. Positively charged residues K, R, and polar H residues are in blue. Negatively charged residues D, E, Q, and the polar N residues are in red. Symbols are used to represent amino acids with peculiar structural properties (red star for proline, black diamond for glycine, square and dotted square for threonine and serine, respectively, which may be either exposed or buried). **d** Structure of the template (human CaMKIIα) and the models (fly VV- and MM-CaMKII). Secondary elements, α-helices, β-sheets, and loops are colored red, yellow, and white, respectively. Residues 280-281 of human CaMKIIα and 281-282 of fly CaMKII are in a ball-and-stick representation. They are colored by atom-type with α carbons in cyan for the human enzyme and in magenta for the fly enzyme.

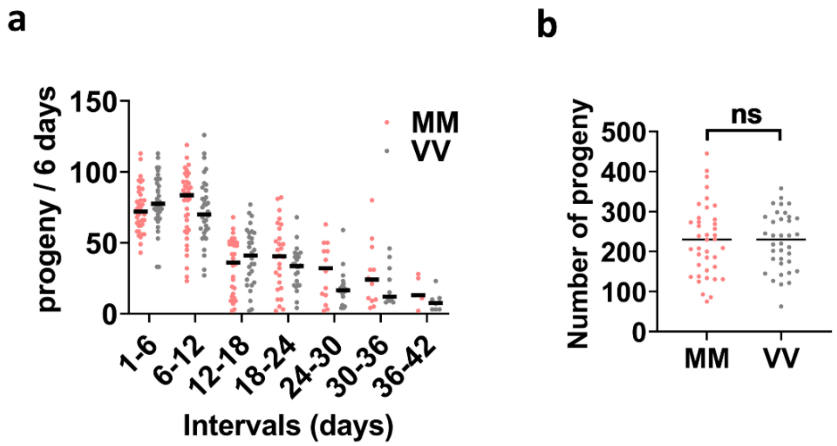

Supplementary Fig. 4 Lifetime fecundity of MM and VV flies. a The number of progenies produced by individual females throughout their reproductive lifespan during six-day intervals. Sidak's multiple comparisons test, two-tailed, were performed between genotypes within each interval and no significant difference was found. **b** Total numbers of progeny produced by individual females, Welch's *t* test, two-tailed. n = 39 for MM and n = 36 for VV flies for both **a** and **b**. Source data are provided in a Source Data file.

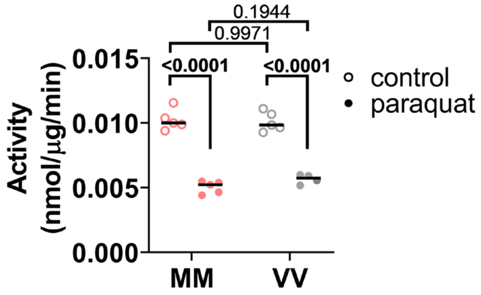

Supplementary Fig. 5 Aconitase activity assay. Flies that were 4-day post eclosure were treated either by 5% sucrose solution or 5% sucrose solution containing 4 mM paraquat for 24 hours before being subjected to aconitase activity assay. Each sample represents the pooled lysate from 15 female and 15 male flies. n = 5 for MM control, MM treated by paraquat, and VV control; n = 4 for VV treated by paraquat; Dunnett's T3 multiple comparisons test, two-tailed. Source data are provided in a Source Data file.

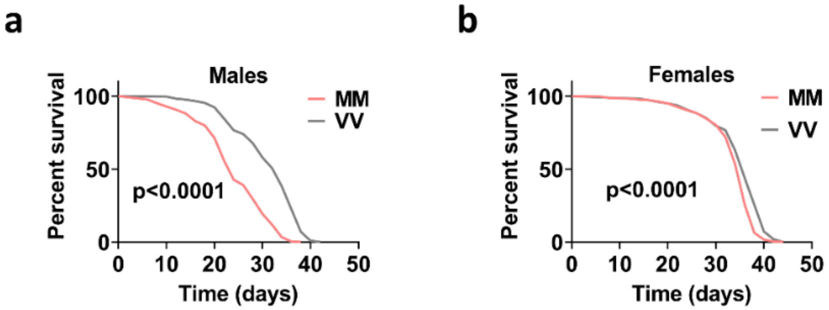

Supplementary Fig. 6 Fly lifespan at 29 °C. a Male and **b** female flies were grouped at the density of 30 flies per vial and monitored for their entire lifespan. n = 269 for MM males, n = 300 for VV males, n = 299 for MM females, and n = 300 for VV females, log-rank test, two-tailed. Source data are provided in a Source Data file.

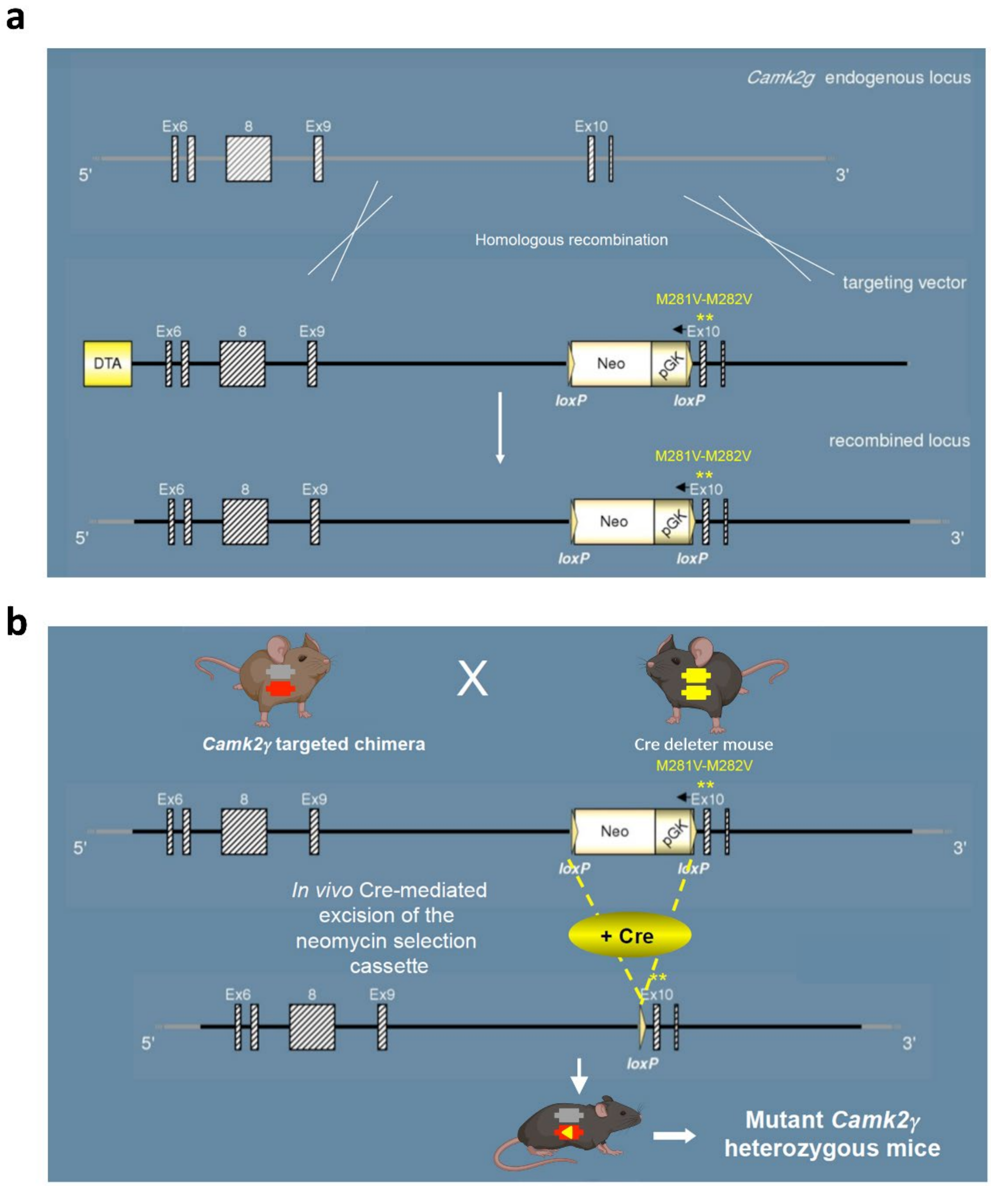

Supplementary Fig. 7 Generation of knock-in *CaMKII*^{VV}/*CaMKII*^{VV} mice. a Schematics of the *Camk2g* endogenous locus (top), targeting vector (middle), and targeted locus (bottom). The MM residues are encoded by exon 10. The gene targeting was carried out on ES cells of C57BL6/n background. **b** Chimeric mice bearing the targeted VV allele were crossed with Cre mice to remove the Neo-pGK cassette between the two LoxP sites. The resulting *CaMKII*^{MM}/*CaMKII*^{VV} heterozygous mice were generated backcrossed to C57BL/6J mice for >7 generations. Diagrams were created with BioRender.com.

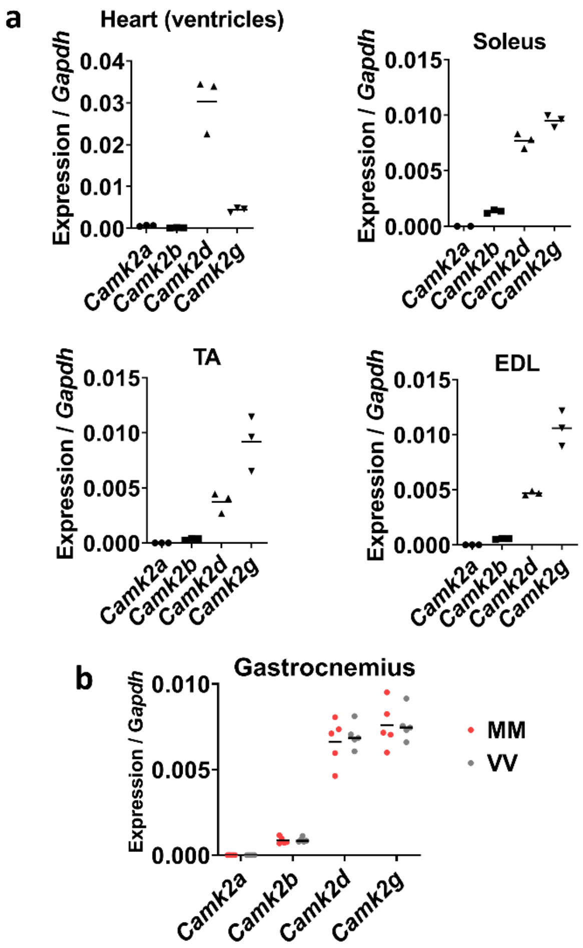

Supplementary Fig. 8 Expression of CaMKII isoforms in representative striated muscles quantified by RT-qPCR. a Expression of CaMKII isoforms in the heart, soleus, TA (tibialis anterior), and EDL (extensor digitorum longus) muscles relative to *Gapdh*. Each tissue was represented by three individual wild type animals. The ratios between *Camk2g* and *Camk2d* vary among different skeletal muscles, but *Camk2g* is consistently expressed at a higher level. **b** RT-qPCR assay for the expression of all four *Camk2* isoforms in gastrocnemius muscles from MM and VV mice; n = 5 for each genotype; no significant difference found between MM and VV muscles for each isoform; Dunn's multiple comparisons tests. In **a** and **b**, horizontal lines indicate means. Source data are provided in a Source Data file.

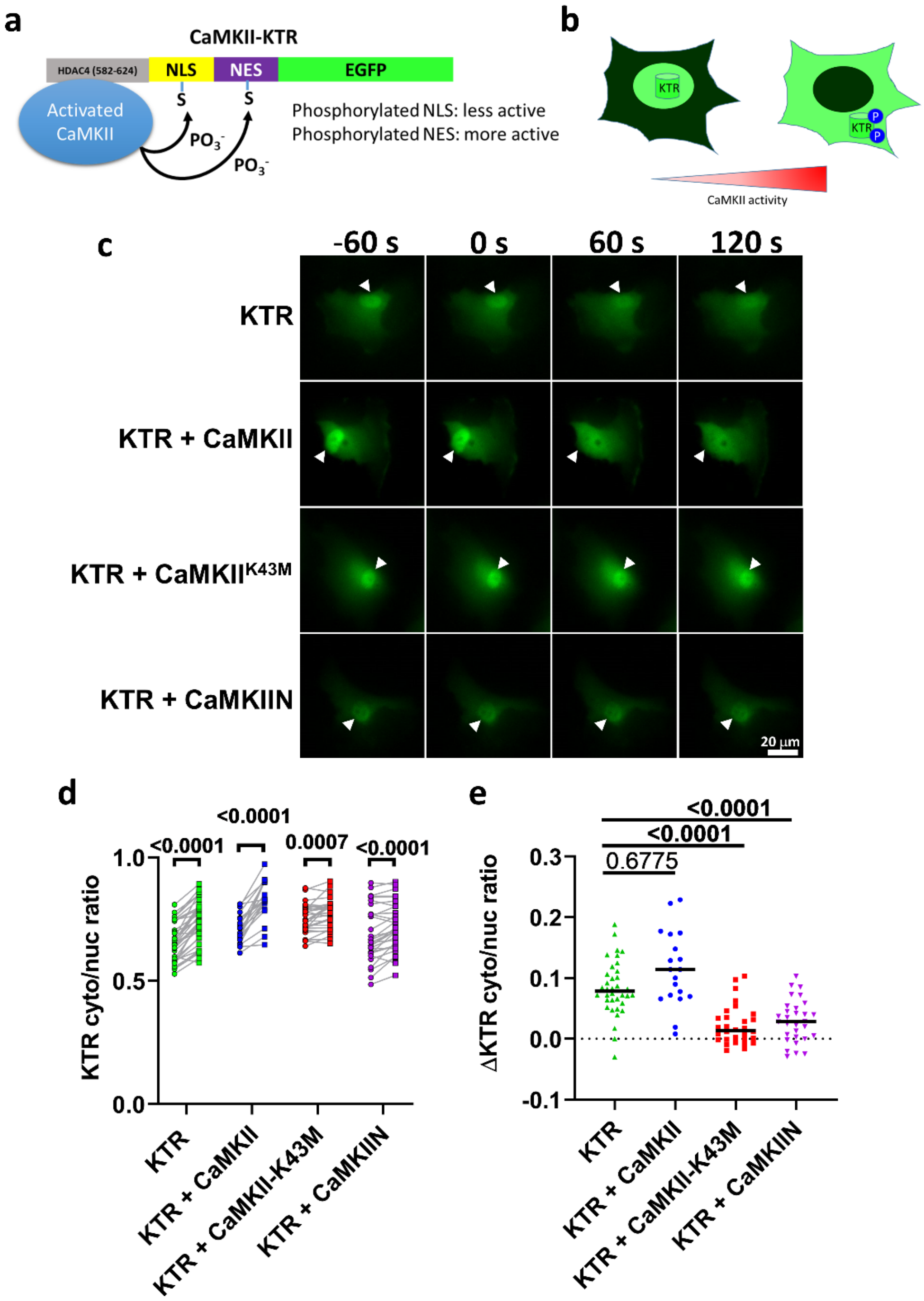

Supplementary Fig. 9 Design and validation of the CaMKII activity reporter, CaMKII-KTR. **a** Schematic of the CaMKII kinase activity translocation reporter CaMKII-KTR (abbreviated as KTR). The N-terminus of the KTR is a well-characterized CaMKII-interacting domain from HDAC4 (AA582-624[4]), followed by a nuclear localization signal (NLS) and a nuclear exporting signal (NES). The high affinity CaMKII substrate consensus sequence (LXRXX**S**V) was built into both the NLS and NES (see Method). The C-terminus of the CaMKII-KTR is an enhanced green fluorescent protein (EGFP). **b** The KTR shuttles between the nucleus and cytosol. Phosphorylation by CaMKII decreases the strength of the NLS while

increases the strength of the NES, resulting in a net translocation of the KTR into the cytosol. The ratio between the cytosolic and nuclear signals of the KTR corresponds to the overall activity of CaMKII inside the cells. **c** Fluorescent images of KTR transfected into RPE-1 cells alone, co-transfected with CaMKII, with kinase-dead $CaMKII^{K43M}$, or with a CaMKII-specific inhibitor CaMKIIN. The arrowheads indicate nuclei. Cells were imaged at time -60 seconds (s), 0, 60 s, and 120 s, and were treated at time 0 with 50 μM of histamine. Treatment with vehicle (medium) did not elicit a response and is not shown. **d** Quantification of cytosolic to nuclear KTR signal ratios in RPE-1 cells as exemplified in **c** immediately before and 60 seconds after the histamine treatment. *P*-values are shown in the graph, paired *t* tests, two-tailed, comparing before and after histamine treatment. **e** Changes in KTR cytosolic to nuclear signal ratios 60 s after histamine treatment compared to time 0 in cells shown in **d**. Horizontal lines indicate the median; *P*-values are shown in the graph, Dunn's multiple comparisons tests, two-tailed. In **d** and **e**, n = 36 KTR transfected cells, n = 19 KTR + CaMKII cells, n = 30 KTR + $CaMKII^{K43M}$ cells, and n = 30 KTR + CaMKIIN cells. Data in **d** and **e** were from at least 2 independent experiments. Source data are provided in a Source Data file.

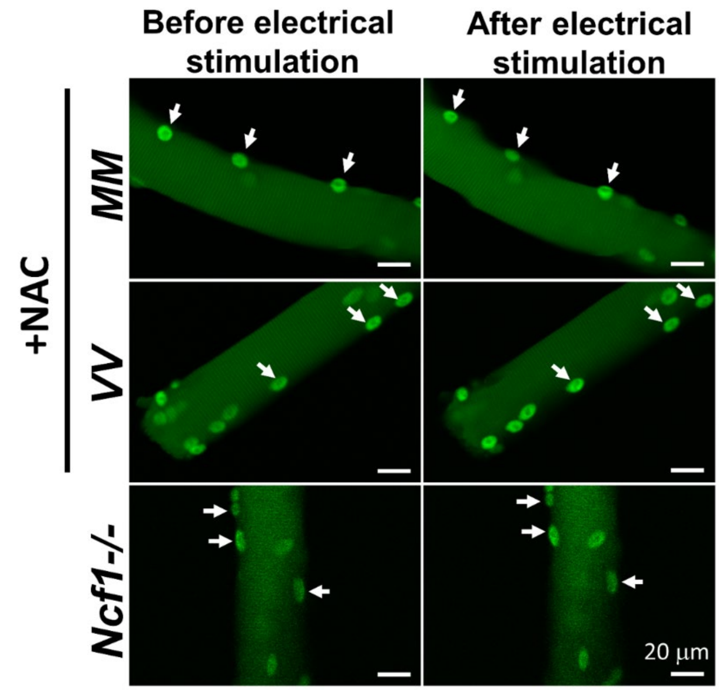

Supplementary Fig. 10 Exemplar confocal images of CaMKII-KTR in MM and VV fibers in the presence of NAC or in *Ncf1*$^{-/-}$ fibers before (left) and after (right) repetitive electrical stimulation. Same stimulation protocol as in Fig 2g, arrows indicate nuclei. Quantification of KTR signal is shown in Fig. 2 **h** and **i**.

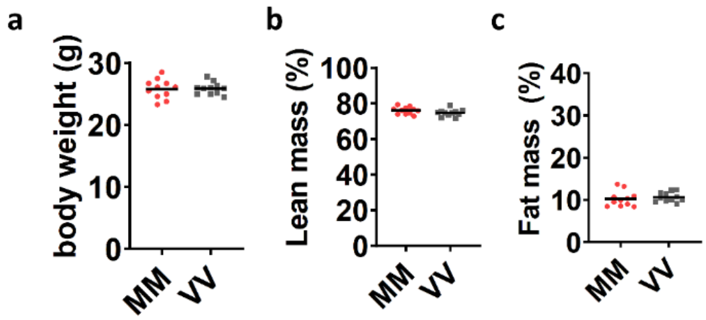

Supplementary Fig. 11 Body composition of MM and VV mice are similar.
a Body weight. **b** Lean mass percentage. **c** Fat mass percentage. N=11 for both MM and VV in **a**, **b,** and **c**. No statistically significant difference was found between genotypes, Welch's *t* test, two-tailed. Source data are provided in a Source Data file.

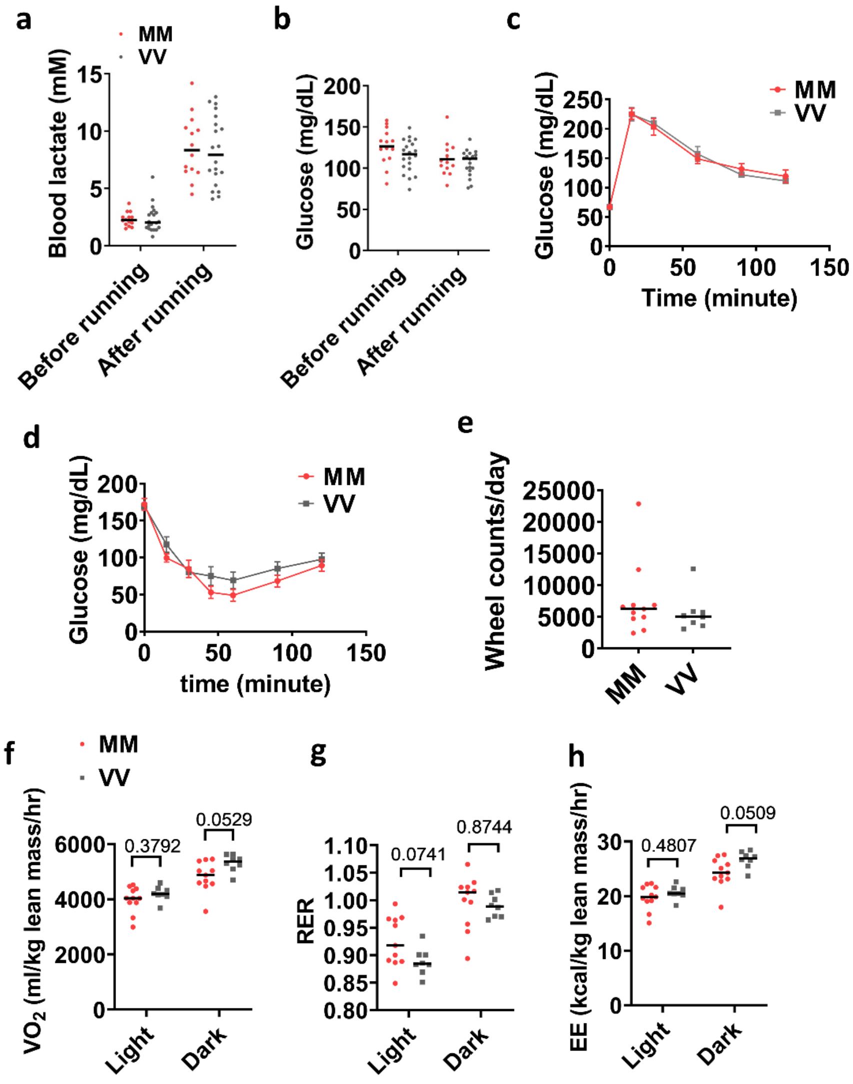

Supplementary Fig. 12 Blood lactate, voluntary running, and metabolism a Lactate concentrations and **b** glucose measured with a drop of blood from tail tips before and immediately after treadmill exercise, n = 14 MM, and n = 20 VV mice, no statistically significant differences were present between genotypes either before or after exercise, Dunnett's T3 multiple comparisons tests. **c** Intraperitoneal glucose tolerance test. Mice were injected with 2 g/kg glucose after overnight (16 hours) fasting. N = 10 for both MM and VV mice. **d** Insulin sensitivity test. Mice were injected with 1 U/kg insulin after food withdraw for 6.5 hours. N = 9 for MM and n = 10 for VV mice. The measure of center in **a-d** are the means. In **c** and **d** error bars are standard errors; no difference between genotypes were found by two-way ANOVA analyses. **e** Counts of average daily wheel rotations during 6 days of running wheel access by individual MM (n = 11) and VV (n = 8) mice, no statistically significant difference was found between genotypes, Mann-Whitney test. **f-h** Mice monitored in **e** were analyzed for **f** oxygen consumption rate normalized to lean body mass, **g** respiratory exchange ratio, and **h** energy expenditure normalized to lean body mass; no significant differences were found between genotypes; *P*-values from two-tailed Dunnett's T3 multiple comparisons tests are shown. Source data are provided in a Source Data file.

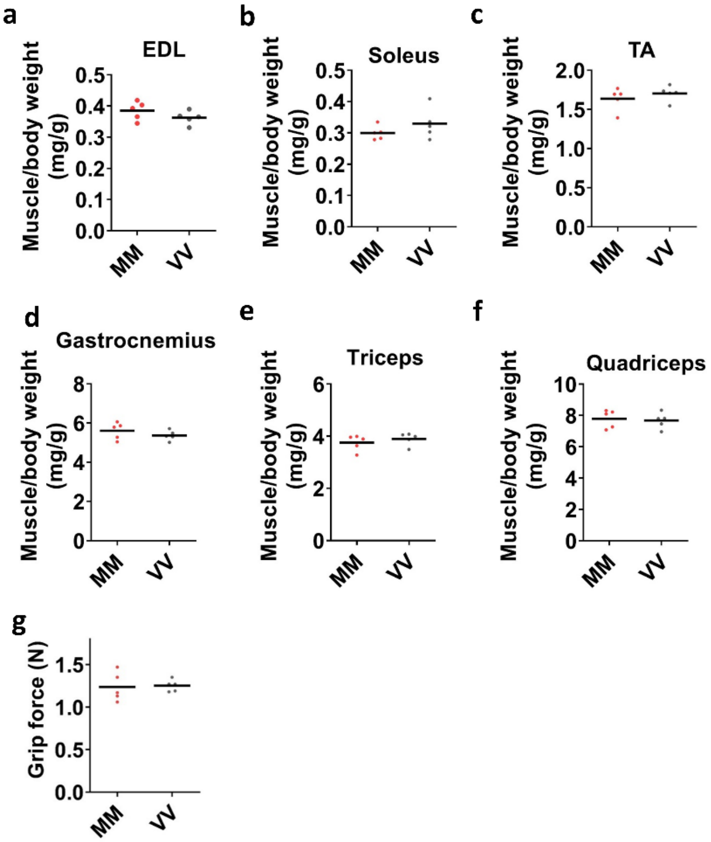

Supplementary Fig. 13 MM (WT) and VV skeletal muscles show no difference in weight and strength. a-f Muscle to body weight ratios of representative skeletal muscles. **a** EDL (extensor digitorum longus). **b** Soleus. **c** TA (tibialis anterior). **d** Gastrocnemius. **e** Triceps. **f** Quadriceps. **g** Grip force of front paws. Horizontal bars indicate means, n = 5 MM, and n = 5 VV mice from **a-g**. Source data are provided in a Source Data file.

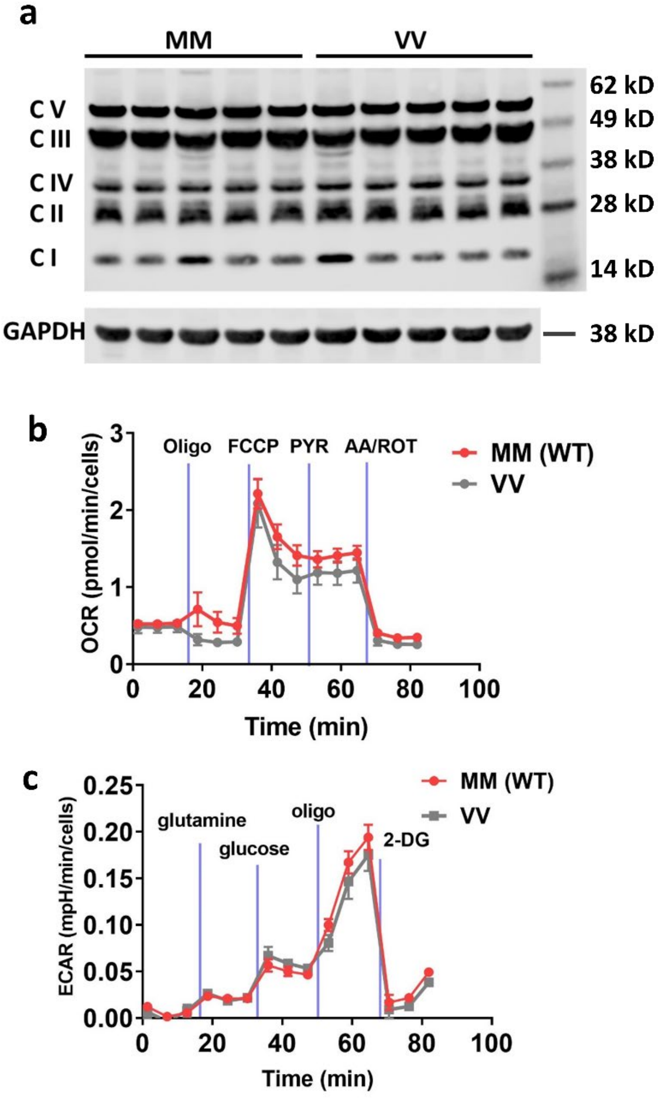

Supplementary Fig. 14 Oxidative and glycolytic capacities of MM and VV muscles are similar. **a** Western blot of gastrocnemius muscle extracts showed no difference in the expression of representative protein subunits from mitochondrial oxidative phosphorylation complexes (n = 5 mice for each genotype). Loading control GAPDH was blotted on the same membrane after stripping the membrane. **b** and **c** Isolated flexor digitorum brevis (FDB) fibers were analyzed for **b** oxygen consumption (OCR) and **c** extracellular acidification (ECAR) rates. Points and error bars are mean ± SEM. No statistically significant differences (Sidak's multiple comparisons tests, two-tailed) were found between MM and VV fibers, n = 5 MM (WT) mice and n = 4 VV mice in **b** and **c**. Source data are provided in a Source Data file.

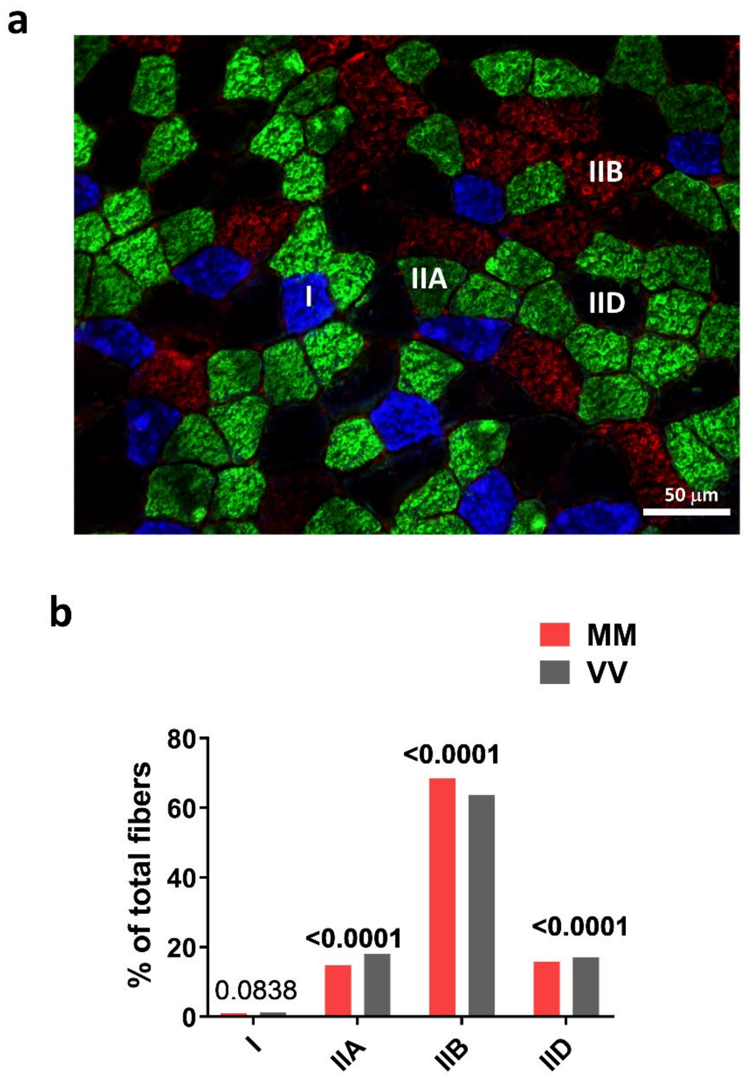

Supplementary Fig. 15 Analysis of skeletal muscle fiber types in MM and VV mice. a An example cross sectional image of quadriceps muscle with immunostaining for myosin heavy chain isoforms I (blue), IIA (green), IIB (red), and IID (black, unstained). Note that this image was chosen to show the staining of all four types of myosin heavy chain isoforms, and the proportions of isoforms in this image do not reflect the entire cross section of the muscle because fiber types are not uniformly distributed throughout the cross-section of the muscles. **b** Quantification of the percentages of fiber types determined by myosin heavy chain isoform expression in MM (n = 5 mice, 1 section per mouse, 20110 fibers counted in total) and VV (n = 5 mice, 1 section per mouse, 22922 fibers counted in total) mice (Fisher's exact test, two-tailed, comparing the same fiber types between MM and VV muscles). All fibers in the entire cross sections of the muscles were counted. Source data are provided in a Source Data file.

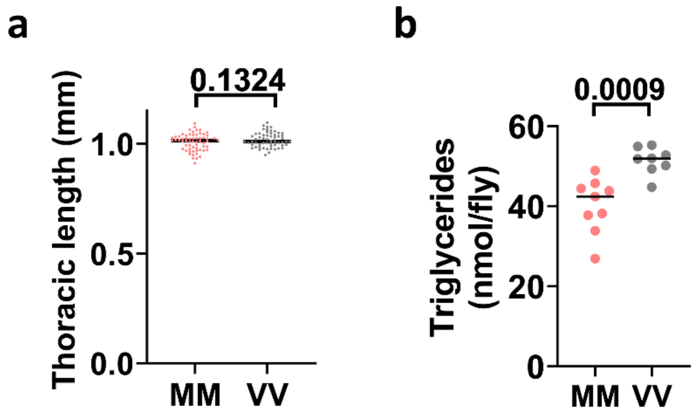

Supplementary Fig. 16 Thoracic length and triglyceride store in flies. a Thoracic length of MM (n = 62) and VV (n = 58) females, Welch's *t* test, two-tailed. **b** Triglyceride content of female MM (n = 9) and VV (n = 8) flies, Welch's *t* test, two-tailed; each sample in **b** represents eight flies that were randomly pooled. Flies in **a** and **b** were the same age as the flies used for climbing tests. Source data are provided in a Source Data file.

Supplementary Table 1 Summary of phylogenetic survey of CaMKII sequences. See Supplementary Data 2 for all sequence alignments.

Groups	Number of sequences surveyed	Number of sequences with MM/CM at loci 281/282	Number of sequences without MM/CM at loci 281/282
Choanoflagellatea	2	0	2
Filasterea: *Capsaspora owczarzaki*	1	0	1
Porifera	2	1	1
Placozoa: *Trichoplax adhaerens*	1	0	1
Cnidaria	14	0	14
Ecdysozoan: *Priapulus caudatus*	1	1	0
Ecdysozoans: Nematoda	7	0	7
Ecdysozoans: Arthropoda	136	2	134
Lophotrochozoans: Mollusca	9	0	9
Echinodermata	2	0	2
Hemichordata: *Saccoglossus kowalevskii*	1	0	1
Cephalochordata	2	0	2
Urochordata: *Ciona intestinalis*	1	0	1
Vertebrata: Cyclostomata	2	2	0
Vertebrata: Chondricthyes	6	6	0
Vertebrata: Actinopterygia	481	454	27[#]
Vertebrata: Actinistia	3	3	0
Vertebrata: Amphibians	19	19	0
Vertebrata: Squamata	35	35	0
Vertebrata: Testudines	15	15	0
Vertebrata: Crocodylia	14	14	0
Vertebrata: Aves	225	225	0
Vertebrata: Mammalia: Monotremata	4	4	0
Vertebrata: Mammalia: Marsupialia	16	16	0
Vertebrata: Mammalia: Placentalia	488	488	0
**All Non-vertebrates**	**179**	**4 (2.23%)**	**175 (97.77%)**
**All Vertebrates**	**1308**	**1281 (97.94%)**	**27 (2.06%)**

[#]Among Actinopterygia (ray-finned fishes), the teleosts possess eight or more copies of CaMKII genes, likely due to at least one extra round of gene/genome duplication. No individual species of the teleost fish have entirely lost the CM/MM module in all CaMKII isoforms; typically, only one CaMKII isoform has lost the module in a single species. The increased redundancy of CaMKII genes in these species might have permitted CM/MM module loss. The alternative modules in teleost fish include VV, IL, MV, MI, KM, and SM. The diversity of these modules, as well as the topology of the teleost tree, suggest that the loss happened multiple times independently.

Supplementary Table 2 Statistics of differential expression analysis by Cuffdiff2.

#Categories	MM (sed) vs. VV (sed)	MM (ex) vs. VV (ex)	MM (sed) vs. MM (ex)	VV (sed) vs. VV (ex)
(1) gene_exp.diff	30529	30599	30598	30618
(2) OK	14628	14689	14825	14628
(3) signif	93	96	743	324
(4) signif.ann	41	46	**582**	**216**
(5) signif.ann.fpkm2	40	41	490	194
(6) signif.ann.fpkm2.logFC1.5	0	3	34	24
(7) signif.ann.fpkm5	33	31	365	157
(8) signif.ann.fpkm5.logFC1.5	0	3	26	21
(9) signif.novel	52	50	161	108
(10) signif.novel.fpkm2	29	27	77	44
(11) signif.novel.fpkm2.logFC1.5	20	25	47	33

#(1) Number of loci; (2) Testable loci; (3) Significant genes (P-value ≤ 0.05, q-value ≤ 0.05); (4) Significant and annotated (known) genes; of which: (5) at least one of FPKM1 and FPKM2 ≥ 2.0; (6) additionally, log2 fold change ≥ 1.5; (7) at least one of FPKM1 and FPKM2 ≥ 5.0; (8) additionally, log2 fold change ≥1.5; (9) Significant and unannotated ('novel') loci; of which: (10) at least one of FPKM1 and FPKM2 ≥ 2.0; (11) additionally, log2 fold change ≥1.5. Statistical tests were performed with Cuffdiff2, which includes correction for multiple tests.

Supplementary Table 3 Alignment statistics of RNA sequencing reads#

Sample	InputReads	Aligned (R1+R2)	(R1+R2) %	Concordant%
VV1 (sed)	41,945,048	39,070,968	93.1	91.90%
VV2 (sed)	42,016,122	38,807,529	92.3	91.00%
VV3 (sed)	46,189,514	41,951,595	90.8	89.40%
VV4 (sed)	43,144,230	39,254,593	90.9	89.60%
VV5 (ex)	47,807,944	42,458,900	88.8	87.30%
VV6 (ex)	38,539,280	35,076,702	91.0	89.60%
VV7 (ex)	49,663,280	44,645,070	89.8	87.90%
VV8 (ex)	49,188,874	44,338,048	90.1	88.80%
MM1 (sed)	46,952,868	43,008,663	91.5	90.20%
MM2 (sed)	46,419,456	41,927,251	90.3	88.90%
MM3 (sed)	41,815,766	39,202,326	93.7	92.50%
MM4 (sed)	48,554,206	45,274,822	93.2	91.90%
MM5 (ex)	42,053,606	38,226,221	90.8	89.50%
MM6 (ex)	39,374,142	36,293,822	92.1	90.90%
MM7 (ex)	50,489,586	46,161,870	91.4	90.20%
MM8 (ex)	47,541,966	43,658,172	91.8	90.60%

Reads were aligned with Tophat2.

Supplementary Table 4 RNA sequencing read classification.

Sample	Aligned	Intergenic	Intron	Exon	Exon-intron	(Ex+Ex-in) %
VV1 (sed)	39,070,968	10,871,032	1,151,817	20,572,924	6,475,195	69.2
VV2 (sed)	38,807,529	10,173,751	1,056,398	20,822,770	6,754,610	71.1
VV3 (sed)	41,951,595	9,850,891	1,100,707	23,295,361	7,704,636	73.9
VV4 (sed)	39,254,593	9,358,135	1,067,585	21,523,146	7,305,727	73.4
VV5 (ex)	42,458,900	9,795,942	1,104,903	23,546,149	8,011,906	74.3
VV6 (ex)	35,076,702	8,350,481	1,020,203	19,380,828	6,325,190	73.3
VV7 (ex)	44,645,070	12,762,540	1,450,013	22,758,083	7,674,434	68.2
VV8 (ex)	44,338,048	12,196,685	1,273,665	23,333,460	7,534,238	69.6
MM1 (sed)	43,008,663	14,389,489	1,223,644	20,909,218	6,486,312	63.7
MM2 (sed)	41,927,251	11,612,157	1,202,400	22,166,915	6,945,779	69.4
MM3 (sed)	39,202,326	9,690,241	1,091,394	21,601,873	6,818,818	72.5
MM4 (sed)	45,274,822	11,798,947	1,183,309	24,310,729	7,981,837	71.3
MM5 (ex)	38,226,221	9,375,367	1,089,510	20,889,204	6,872,140	72.6
MM6 (ex)	36,293,822	9,136,213	1,069,954	19,752,989	6,334,666	71.9
MM7 (ex)	46,161,870	12,957,951	1,406,060	24,129,718	7,668,141	68.9
MM8 (ex)	43,658,172	12,897,911	1,309,884	22,353,997	7,096,380	67.5

Supplementary References

1 Callebaut, I. *et al.* Deciphering protein sequence information through hydrophobic cluster analysis (HCA): current status and perspectives. *Cellular and Molecular Life Sciences (CMLS)* **53**, 621-645, doi:10.1007/s000180050082 (1997).

2 Lemesle-Varloot, L. *et al.* Hydrophobic cluster analysis: procedures to derive structural and functional information from 2-D-representation of protein sequences. *Biochimie* **72**, 555-574, doi:10.1016/0300-9084(90)90120-6 (1990).

3 Gaboriaud, C., Bissery, V., Benchetrit, T. & Mornon, J. P. Hydrophobic cluster analysis: An efficient new way to compare and analyse amino acid sequences. *FEBS Letters* **224**, 149-155, doi:10.1016/0014-5793(87)80439-8 (1987).

4 Backs, J., Song, K., Bezprozvannaya, S., Chang, S. & Olson, E. N. CaM kinase II selectively signals to histone deacetylase 4 during cardiomyocyte hypertrophy. *The Journal of Clinical Investigation* **116**, 1853-1864, doi:10.1172/JCI27438 C2 - PMC1474817 (2006).
