# Supplementary material for: CaMKII oxidation is a critical performance/disease trade-off acquired at the dawn of vertebrate evolution
Source: Nat Commun. 2021 May 26;12:3175. doi: 10.1038/s41467-021-23549-3 (PMC8155201; doi:10.1038/s41467-021-23549-3)
Supplement: Supplementary file 3 — Description of Additional Supplementary Files [file 41467_2021_23549_MOESM3_ESM.pdf]

Description of Additional Supplementary Files

File Name: Supplementary Data 1
Description: List of candidate genes screened by RT-qPCR in MM and VV mouse mast cells, related to Fig. 4a.

File Name: Supplementary Data 2
Description: Sequence alignments underlying Supplementary Table 1.

File Name: Supplementary Data 3
Description: Primer validation data provided by Bio-Rad for custom designed PrimePCRTM assay plates for Fig. 4a.

File Name: Supplementary Movie 1
Description: Examples of categorical cardiac performance of flies.
