# Supplementary material for: CaMKII oxidation is a critical performance/disease trade-off acquired at the dawn of vertebrate evolution
Source: Nat Commun. 2021 May 26;12:3175. doi: 10.1038/s41467-021-23549-3 (PMC8155201; doi:10.1038/s41467-021-23549-3)
Supplement: Supplementary file 6 — Supplementary Data 3 [file 41467_2021_23549_MOESM6_ESM.zip › Bio-Rad PrimePCR plate validation data/Tirap_qMmuCID0019793_Validation_Data.pdf.pdf]

PrimePCR™ Assay Validation Report

Gene Information

**Gene Name**	toll-interleukin 1 receptor (TIR) domain-containing adaptor protein
**Gene Symbol**	Tirap
**Organism**	Mouse
**Gene Summary**	Description Not Available
**Gene Aliases**	AA407980, C130027E04Rik, Mal, Tlr4ap, Wyatt
**RefSeq Accession No.**	NC_000075.6, NT_039472.8
**UniGene ID**	Mm.23987
**Ensembl Gene ID**	ENSMUSG00000032041
**Entrez Gene ID**	117149

Assay Information

**Unique Assay ID**	qMmuCID0019793
**Assay Type**	SYBR® Green
**Detected Coding Transcript(s)**	ENSMUST00000175765, ENSMUST00000176531, ENSMUST00000177129, ENSMUST00000176685, ENSMUST00000177052
**Amplicon Context Sequence**	CCTGCTGACCATGGCCTTCAACAGTCTTCCTAGGTCTTGGTGTCTCCATTGTTCT GGATGCAGGTTACACAAGAAAGCCTGTGAAAAGTGG
**Amplicon Length (bp)**	71
**Chromosome Location**	9:35191648-35199262
**Assay Design**	Intron-spanning
**Purification**	Desalted

Validation Results

**Efficiency (%)**	97
**R^2**	0.9996
**cDNA Cq**	23.69
**cDNA Tm (Celsius)**	78.5
**gDNA Cq**	
**Specificity (%)**	100

Information to assist with data interpretation is provided at the end of this report.

PrimePCR™ Assay Validation Report

Tirap, Mouse

Amplification Plot

Amplification of cDNA generated from 25 ng of universal reference RNA

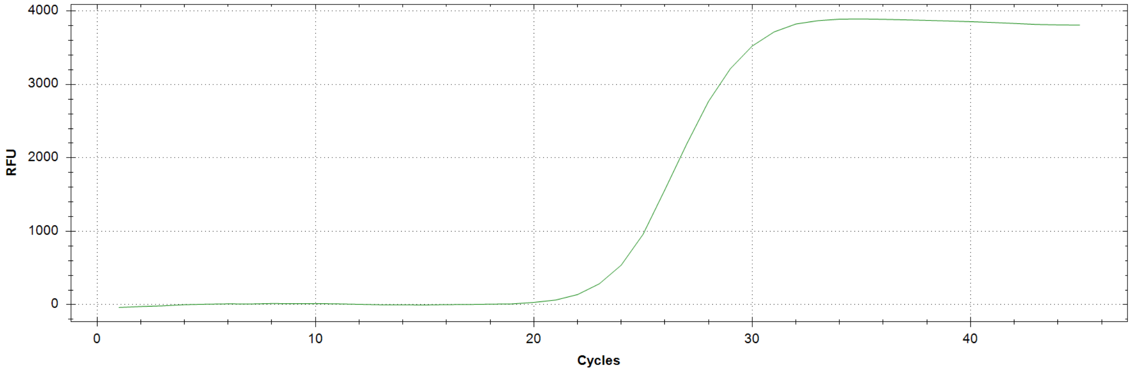

Melt Peak

Melt curve analysis of above amplification

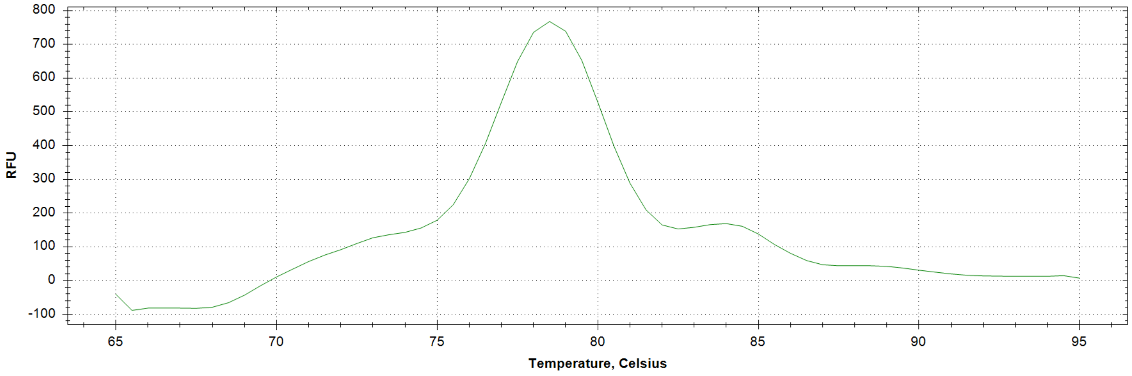

Standard Curve

Standard curve generated using 20 million copies of template diluted 10-fold to 20 copies

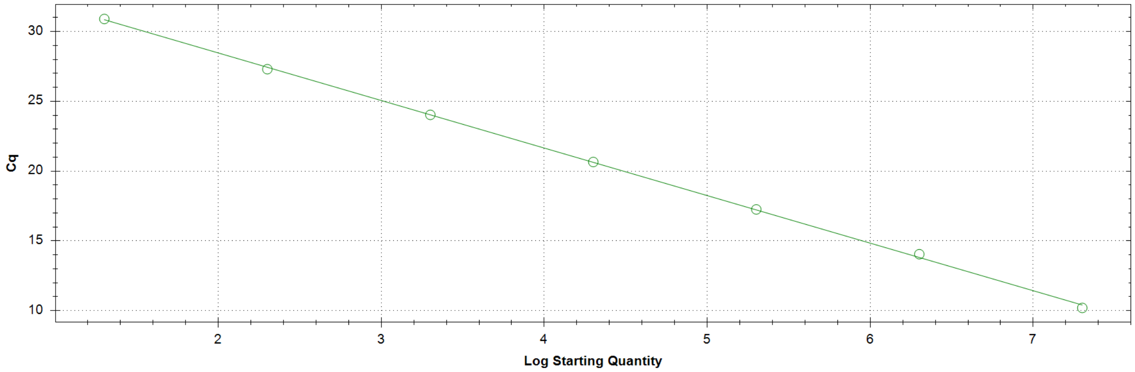

PrimePCR™ Assay Validation Report

Products used to generate validation data

**Real-Time PCR Instrument**	CFX384 Real-Time PCR Detection System
**Reverse Transcription Reagent**	iScript™ Advanced cDNA Synthesis Kit for RT-qPCR
**Real-Time PCR Supermix**	SsoAdvanced™ SYBR® Green Supermix
**Experimental Sample**	qPCR Mouse Reference Total RNA

Data Interpretation

**Unique Assay ID**	This is a unique identifier that can be used to identify the assay in the literature and online.
**Detected Coding Transcript(s)**	This is a list of the Ensembl transcript ID(s) that this assay will detect. Details for each transcript can be found on the Ensembl website at www.ensembl.org.
**Amplicon Context Sequence**	This is the amplicon sequence with additional base pairs added to the beginning and/or end of the sequence. This is in accordance with the minimum information for the publication of real-time quantitative PCR experiments (MIQE) guidelines. For details, please refer to the following publication, "Primer Sequence Disclosure: A Clarification of the MIQE Guidelines" (Bustin et al 2011).
**Chromosome Location**	This is the chromosomal location of the amplicon context sequence within the genome.
**Assay Design**	Exonic: Primers sit within the same exon in the mRNA transcript and can potentially co-amplify genomic DNA. If performing gene expression analysis, it is suggested that the samples be treated with a DNase to eliminate potential unwanted signal from contaminating genomic DNA.<br><br>Exon-exon junction: One primer sits on an exon-exon junction in mRNA. When performing gene expression analysis, this design approach will prevent unwanted signal from contaminating genomic DNA.<br><br>Intron-spanning: Primers sit within different exons while spanning a large intron in the mRNA (intron is greater than 750bp). When performing gene expression analysis, this design approach should limit potential unwanted signal from contaminating genomic DNA.<br><br>Small intron-spanning: Primers sit within different exons with a short intron in between (intron is smaller than 750bp). Small introns may not prevent unwanted signal from contaminating genomic DNA.
**Efficiency**	Assay efficiency was determined using a seven-point standard curve from 20 copies to 20 million copies. While an efficiency of 100% represents a perfect doubling of template at every cycle and is ideal, typical ranges of good assay efficiency are between 90-110%. For difficult targets, assay efficiency outside of this range are accepted and reported accordingly.
**R^2**	The R^2 represents the linearity of the standard curve and how well the standard curve data points fit the linear regression line. Acceptable values are >0.98.

**cDNA Cq**	Cq value obtained from 25ng of cDNA transcribed from universal RNA when performing wet-lab validation of the assay.<br><br>Note: Not all genes will be expressed at a detectable level in the universal RNA sample.
**cDNA Tm**	Melting temperature of the amplicon when running a melt curve analysis.
**gDNA Cq**	Cq value obtained when running the assay with 2.5ng of genomic DNA. This is more than a moderate level of genomic DNA contamination. Intron-spanning and exon-exon junction assay designs can minimize or eliminate genomic DNA detection.<br><br>Note: Genomic DNA contamination is often present at variable levels. If concerned about genomic DNA contamination, the genomic DNA contamination control assay is recommended to run with your sample to determine if genomic DNA levels are sufficient to negatively impact results.
**Specificity**	This value is the percent of specific amplicon reads as measured by next generation sequencing (NGS). While 100% specificity is desirable, small decreases in specificity (<1%) can be due to NGS read errors. More significant reductions are likely due to co-amplification of homologous regions.<br><br>Note: Since gene expression can be cell type and condition specific, the exact level and impact of co-amplification in a given sample is impossible to predict. If co-amplification is detected, it should be taken into consideration and reported when analyzing gene expression results.
