# Supplementary material for: CaMKII oxidation is a critical performance/disease trade-off acquired at the dawn of vertebrate evolution
Source: Nat Commun. 2021 May 26;12:3175. doi: 10.1038/s41467-021-23549-3 (PMC8155201; doi:10.1038/s41467-021-23549-3)
Supplement: Supplementary file 8 — Reporting Summary [file 41467_2021_23549_MOESM8_ESM.pdf]

nature research

Corresponding author(s): Mark E. Anderson
Qinchuan Wang

Last updated by author(s): May 3, 2021

Reporting Summary

Nature Research wishes to improve the reproducibility of the work that we publish. This form provides structure for consistency and transparency in reporting. For further information on Nature Research policies, see our Editorial Policies and the Editorial Policy Checklist.

Statistics

For all statistical analyses, confirm that the following items are present in the figure legend, table legend, main text, or Methods section.

n/a	Confirmed	
☐	☒	The exact sample size (*n*) for each experimental group/condition, given as a discrete number and unit of measurement
☐	☒	A statement on whether measurements were taken from distinct samples or whether the same sample was measured repeatedly
☐	☒	The statistical test(s) used AND whether they are one- or two-sided<br>*Only common tests should be described solely by name; describe more complex techniques in the Methods section.*
☒	☐	A description of all covariates tested
☐	☒	A description of any assumptions or corrections, such as tests of normality and adjustment for multiple comparisons
☐	☒	A full description of the statistical parameters including central tendency (e.g. means) or other basic estimates (e.g. regression coefficient) AND variation (e.g. standard deviation) or associated estimates of uncertainty (e.g. confidence intervals)
☐	☒	For null hypothesis testing, the test statistic (e.g. *F*, *t*, *r*) with confidence intervals, effect sizes, degrees of freedom and *P* value noted<br>*Give P values as exact values whenever suitable.*
☒	☐	For Bayesian analysis, information on the choice of priors and Markov chain Monte Carlo settings
☒	☐	For hierarchical and complex designs, identification of the appropriate level for tests and full reporting of outcomes
☒	☐	Estimates of effect sizes (e.g. Cohen's *d*, Pearson's *r*), indicating how they were calculated

Our web collection on statistics for biologists contains articles on many of the points above.

Software and code

Policy information about availability of computer code

Data collection	CellSens Imaging Software V2.3 64 bit, Olympus Scientific Solutions Americas Corp. Waltham, MA, USA<br>Fluoview500, Olympus Scientific Solutions Americas Corp. Waltham, MA, USA<br>AIM-LSM, Carl Zeiss Microscopy, LLC. White Plains, NY, USA<br>LabView version 8.5, National Instruments, Austin, TX<br>OneTouch Ultra 2 glucometer, Lifescan, Inc.<br>Lactate Plus lactate meter, Nova Biomedical<br>Sony - HDRCX405 HD Video Recording Handycam Camcorder
Data analysis	Fiji ImageJ, ImageJ v1.53c (Schindelin, J.; Arganda-Carreras, I. & Frise, E. et al. (2012), "Fiji: an open-source platform for biological-image analysis", Nature methods 9(7): 676-682, PMID 22743772, doi:10.1038/nmeth.2019<br><br>Origin(Pro)8, OriginLab Corporation, Northampton, MA, USA.<br><br>GraphPad Prism 8, GraphPad Software, Inc.<br><br>CellProfiler 3.1.8, McQuin C, Goodman A, Chernyshev V, Kamentsky L, Cimini BA, Karhohs KW, Doan M, Ding L, Rafelski SM, Thirstrup D, Wiegraebe W, Singh S, Becker T, Caicedo JC, Carpenter AE (2018). CellProfiler 3.0: Next-generation image processing for biology. PLoS Biol. 16 (7):e2005970 / doi. PMID: 29969450 (Research article)<br><br>Mega-X v10.1.8 (https://www.megasoftware.net/)

Bio-Rad CFX Manager 3.1

FlowJo software v10 (Tree Star, Ashland, OR).

Microsoft Excel

For manuscripts utilizing custom algorithms or software that are central to the research but not yet described in published literature, software must be made available to editors and reviewers. We strongly encourage code deposition in a community repository (e.g. GitHub). See the Nature Research guidelines for submitting code & software for further information.

Data

Policy information about availability of data

All manuscripts must include a data availability statement. This statement should provide the following information, where applicable:

- Accession codes, unique identifiers, or web links for publicly available datasets
- A list of figures that have associated raw data
- A description of any restrictions on data availability

Raw RNA sequencing data and the gene-expression matrix are available in the Gene Expression Omnibus (GEO) under accession number GSE132520. Go to https://www.ncbi.nlm.nih.gov/geo/query/acc.cgi?acc=GSE132520
All other source data are included in source data files.
Publicly available data used in this study were retrieved as follows:
Most CaMKII orthologues listed in Supplementary Fig.1 and Supplementary Table 1 were identified in the Interpro database (http://www.ebi.ac.uk/interpro/entry/IPR013543/taxonomy). Additional sequences were uncovered by BLAST in the NCBL nucleotide database and translated into proteins (https://blast.ncbi.nlm.nih.gov/Blast.cgi). All sequence files with accession numbers are available in Supplementary data 2.
Human mast cell transcriptome profiling dataset GSE125887 was retrieved from GEO (https://www.ncbi.nlm.nih.gov/geo/query/acc.cgi?acc=GSE125887).
Transcriptome changes of Drosophila melanogaster in response to paraquat treatment were obtained from Supplementary Table 9 of Brown, J. B. et al. Diversity and dynamics of the Drosophila transcriptome. Nature 512, 393-399, doi:10.1038/nature12962 (2014).

Field-specific reporting

Please select the one below that is the best fit for your research. If you are not sure, read the appropriate sections before making your selection.

[x] Life sciences [ ] Behavioural & social sciences [ ] Ecological, evolutionary & environmental sciences

For a reference copy of the document with all sections, see nature.com/documents/nr-reporting-summary-flat.pdf

Life sciences study design

All studies must disclose on these points even when the disclosure is negative.

Sample size

No statistical calculation was used to determine sample size. Sample sizes were considered adequate based on relevant published literature. For example: for mouse exercise tests fewer than 20 mice were used in Marcaletti, S., Thomas, C. & Feige, J. N. Exercise Performance Tests in Mice. Current Protocols in Mouse Biology, doi:10.1002/9780470942390.mo100160 (2011).

For mouse metabolic tests: Shah, R. et al. Metabolic Effects of CX3CR1 Deficiency in Diet-Induced Obese Mice. Plos One 10, e0138317, doi:10.1371/journal.pone.0138317 C2 - PMC4579121 (2015).

For muscle fiber experiments, Shen, T. et al. DNA binding sites target nuclear NFATc1 to heterochromatin regions in adult skeletal muscle fibers. Histochem Cell Biol 134, 387-402, doi:10.1007/s00418-010-0744-4 (2010).

For fly lifespan experiment: Linford, N. J., Bilgir, C., Ro, J. & Pletcher, S. D. Measurement of Lifespan in Drosophila melanogaster. Journal of Visualized Experiments, doi:10.3791/50068 (2013).

For fly cardiac function test: Konstantinidis, K. et al. MICAL1 constrains cardiac stress responses and protects against disease by oxidizing CaMKII. Journal of Clinical Investigation 130, 4663-4678, doi:10.1172/jci133181 (2020)

Additional relevant references are cited in the manuscript where conventions of sample sizes in the field can be seen.

Data exclusions

Three out of 90 genes in Figure 4b failed to be amplified by RT-qPCR from mast cell cDNA and were excluded from the pricipal analysis in Figure 4b.

Replication

Most experiments were performed at least three times, and all replications were successful. Exceptions are the following:

Fig. 1d, e, and supplementary Fig. 6 Fly lifespan experiments were performed independently at 25 degrees Celsius and 29 degrees Celsius with large sample sizes, and both conditions support the same conclusion. All individual vials housing 30 flies each showed a consistent mortality difference between genotypes. No additional replication lifespan experiments were performed.

Fig. 3 and 4b RNA sequencing of mouse skeletal muscles and mast cell 384-well PrimePCR qRT-PCR analyses were performed once using pre-optimized protocols and pre-determined sample size; no replications were attempted for either experiment according to the convention in the field and costs considerations.

Fig. 4c. Paraquat treatment and RT-qPCR of flies were performed twice.

Supplementary Fig. 4 Fecundity tests were performed twice.

Randomization

All animals and cells were randomly assigned to the experimental groups.

Blinding

Investigators were blinded to groups during data collection and analysis except for the following:
Fly paraquat treatment, lifespan tests, and climbing tests. These tests were performed by having an equal number of randomly assigned MM and VV flies in parallel in the same experiments under identical conditions; thus, blinding was not considered.

Reporting for specific materials, systems and methods

We require information from authors about some types of materials, experimental systems and methods used in many studies. Here, indicate whether each material, system or method listed is relevant to your study. If you are not sure if a list item applies to your research, read the appropriate section before selecting a response.

Materials & experimental systems

n/a	Involved in the study	
☐	☒	Antibodies
☐	☒	Eukaryotic cell lines
☒	☐	Palaeontology and archaeology
☐	☒	Animals and other organisms
☒	☐	Human research participants
☒	☐	Clinical data
☒	☐	Dual use research of concern

Methods

n/a	Involved in the study	
☒	☐	ChIP-seq
☐	☒	Flow cytometry
☒	☐	MRI-based neuroimaging

Antibodies

Antibodies used

Total OXPHOS Rodent WB Antibody Cocktail from Abcam (ab110413, 1:1000 dilution).
GAPDH (D16H11) XP® Rabbit mAb (Cell Signalling, #5174, 1:2000 dilution).
BA-F8-c (myosin heavy chain, slow, 1:50), SC-71-c (Myosin Heavy Chain Type IIA, 1:600), BF-F3-c (Myosin Heavy Chain Type IIB, 1:100), 6H1-s (myosin heavy chain, fast, IIX 1:500) were obtained from Developmental Studies Hybridoma Bank (University of Iowa).
1 μg/mL anti-OVA IgE (clone E-C1, Chondrex Inc)
10 ng/mL of mouse recombinant IL-3 (BioLenged, 575908)
anti-mouse CD16/32 (clone 93, BioLegend) at 10 μg/mL
cKit/CD117 (APC, 17-1171-812, eBioscience, 1:200)
FcεRI (FITC, 134305, Biolegend, 1:100)
LAMP-1/CD107a (PE, 12-1071-81, eBioscience, 1:100).

Validation

ab110413 oxPhos (https://www.abcam.com/total-oxphos-rodent-wb-antibody-cocktail-ab110413.html).
GAPDH Cell Signaling #5174 (https://www.cellsignal.com/products/primary-antibodies/gapdh-d16h11-xp-rabbit-mab/5174).
Validation of BA-F8-c (myosin heavy chain, slow, 1:50), SC-71-c (Myosin Heavy Chain Type IIA, 1:600), BF-F3-c (Myosin Heavy Chain Type IIB, 1:100), 6H1-s (myosin heavy chain, fast, IIX 1:50) were obtained from Developmental Studies Hybridoma Bank (University of Iowa) for muscle fiber typing in mouse was detailed in Bloemberg, D. & Quadrilatero, J. Rapid determination of myosin heavy chain expression in rat, mouse, and human skeletal muscle using multicolor immunofluorescence analysis. PLoS One 7, e35273, doi:10.1371/journal.pone.0035273 (2012).
anti-OVA IgE (clone E-C1) (https://www.chondrex.com/products/mouse-anti-ova-monoclonal-ige-antibody-e-c-)
10 ng/mL of mouse recombinant IL-3 (BioLenged, 575908), https://www.biolegend.com/en-us/products/recombinant-mouse-il-13-carrier-free-5655?GroupID=GROUP575
anti-mouse CD16/32 (clone 93, BioLegend), https://www.biolegend.com/en-us/products/purified-anti-mouse-cd16-32-antibody-190
cKit/CD117 (APC, 17-1171-812, eBioscience), https://www.thermofisher.com/antibody/product/CD117-c-Kit-Antibody-clone-2B8-Monoclonal/17-1171-81
FcεRI (FITC, 134305, Biolegend), https://www.biolegend.com/ja-jp/products/fitc-anti-mouse-fcepsilonrialpha-antibody-5949
LAMP-1/CD107a (PE, 12-1071-81, eBioscience), https://www.thermofisher.com/antibody/product/CD107a-LAMP-1-Antibody-clone-eBio1D4B-1D4B-Monoclonal/12-1071-82

Eukaryotic cell lines

Policy information about cell lines

Cell line source(s)	RPE-1 cells were obtained from ATCC® (CRL-4000™)
Authentication	Cell line purchased from ATCC but not authenticated by us
Mycoplasma contamination	Not tested
Commonly misidentified lines (See ICLAC register)	No commonly misidentified cell lines were used in the study.

Animals and other organisms

Policy information about studies involving animals; ARRIVE guidelines recommended for reporting animal research

Laboratory animals

All mice used in this study were backcrossed to C57BL/6J background for minimal of 7 generations.

Only male mice were used in this study.

Mice used to prepare bone marrow derived mast cells were between 6 and 8 weeks of age. All other mice used in these studies were between 10-15 weeks of age.

The CaMKIIdeltaVV mice were generated and characterized by us (reference 13 of the manuscript, Luo, M. et al. Diabetes increases mortality after myocardial infarction by oxidizing CaMKII. J Clin Invest 123, 1262-1274, doi:10.1172/JCI65268 (2013).)

The CaMKIIgammaVV mice were generated by GenOway as described

The Ncf1-/- mice were obtained from Jackson Laboratory (Cat #004742) and maintained on a C57BL/6J background

All fly experiments reported were performed with flies back crossed to iso31 for 5 generations.

Wild animals	This study did not involve wild animals.
Field-collected samples	This study did not involve samples collected from the field.
Ethics oversight	All animal handling procedures were in accordance with National Institutes of Health guidelines and were approved by the Institutional Animal Care and Use Committees of Johns Hopkins University School of Medicine.

Note that full information on the approval of the study protocol must also be provided in the manuscript.

Flow Cytometry

Plots

Confirm that:

- [x] The axis labels state the marker and fluorochrome used (e.g. CD4-FITC).
- [x] The axis scales are clearly visible. Include numbers along axes only for bottom left plot of group (a 'group' is an analysis of identical markers).
- [x] All plots are contour plots with outliers or pseudocolor plots.
- [x] A numerical value for number of cells or percentage (with statistics) is provided.

Methodology

Sample preparation	For FACS analysis, single-cell suspensions (1x10^5) were blocked with anti-mouse CD16/32 (clone 93, BioLegend) at 10 µg/mL in PBS for 20 min, and then stained with fluorescently labeled antibodies against mast cell surface markers or activation markers for 30 min on ice. Antibodies against mouse antigens were: cKit/CD117 (APC, 17-1171-812, eBioscience, 1:200), FcεRI (FITC, 134305, Biolegend, 1:100), and LAMP-1/CD107a (PE, 12-1071-81, eBioscience, 1:100). FACS analysis was performed on a FACSCalibur flow cytometer (BD Biosciences) using FlowJo software (Tree Star, Ashland, OR).
Instrument	BD Accuri C6 Plus flow cytometer
Software	Flow Jo v10
Cell population abundance	Mast cells are over 98% in our preparations.
Gating strategy	Gating of single cells using FSC/ W and SSC/W and exclusion of dead cells with the LIVE/DEAD™ Fixable Far-Red Dead Cell

Stain (ThermoFisher).
The flow cytometry assay was for quality control purpose. We did not include data in our manuscript.

☐ Tick this box to confirm that a figure exemplifying the gating strategy is provided in the Supplementary Information.
